# Co‐Delivery of Varicella Zoster Virus gE and gI Dual‐Antigen mRNA Vaccine With CpG‐ODN Elicits Robust Immunity

**DOI:** 10.1002/mco2.71117

**Published:** 2026-11-01

**Authors:** Lingyan Cui, Sibo Zhang, Wenhui Xue, Hongjing Liu, Hong Wang, Hai Li, Yarong Zeng, Huizheng Zhang, Yaohan Li, Minqing Hong, Lin Chen, Jun Zhang, Ying Gu, Shaowei Li, Tingting Li, Ningshao Xia

**Affiliations:** ^1^ State Key Laboratory of Vaccines for Infectious Diseases, Xiang An Biomedicine Laboratory, School of Public Health, School of Life Sciences, Xiamen University Xiamen China; ^2^ National Institute of Diagnostics and Vaccine Development in Infectious Diseases, Collaborative Innovation Center of Biologic Products, National Innovation Platform for Industry‐Education Integration in Vaccine Research, the Research Unit of Frontier Technology of Structural Vaccinology of Chinese Academy of Medical Sciences, Xiamen University Xiamen China

**Keywords:** CpG‐ODN 1018, glycoprotein E/glycoprotein I, herpes zoster, mRNA vaccine, varicella zoster virus

## Abstract

Herpes zoster (HZ), caused by reactivation of latent varicella zoster virus (VZV), remains a growing health burden. Developing new HZ vaccines with high efficacy and improved tolerability could address limitations of current vaccines and increase vaccination uptake and completion rates. To identify an optimal antigen design, we systematically evaluated multiple mRNA candidates based on VZV glycoprotein E (gE) and I (gI). In mice, membrane‐localized gE induced the strongest and most durable antibody responses, whereas inclusion of gI markedly enhanced T‐cell responses. Co‐delivery of membrane‐localized gE and gI (gE+gI Lmix) elicited the most robust cellular responses, outperforming both single‐antigen and fusion constructs. We therefore selected gE+gI Lmix for further optimization with CpG oligodeoxynucleotide (CpG‐ODN) adjuvants. In both naïve and VZV‐primed mice, CpG‐ODN 1018 increased neutralizing antibody titers and significantly enhanced antigen‐specific CD4^+^ and CD8^+^ IFN‐γ^+^ T‐cell responses, with a Th1‐biased profile. The resulting humoral and cellular responses matched or exceeded those induced by Shingrix in mice. Safety assessments revealed no distinct systemic toxicity or histopathological abnormalities following CpG‐ODN 1018 administration. Together, these findings identify dual‐antigen gE/gI mRNA vaccination, particularly in combination with CpG‐ODN 1018, as a promising strategy for HZ prevention, with enhanced cellular immunogenicity and an acceptable preclinical safety profile.

## Graphical abstract

The gE+gI Lmix+CpG‐ODN 1018 vaccine was formulated by combining gE and gI mRNA/LNPs with the TLR9 agonist adjuvant CpG‐ODN 1018. This formulation induces robust humoral and cellular immunity while maintaining a favorable safety profile and physiological stability in mice.

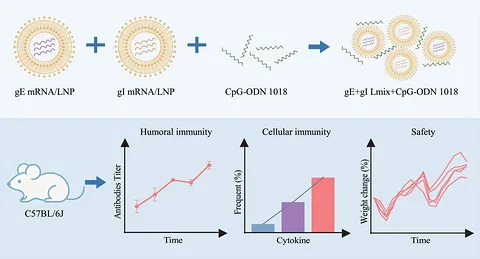

## 1 Introduction

Varicella zoster virus (VZV), a human alpha herpesvirus, causes varicella (chickenpox) during primary infection and establishes lifelong latency in sensory ganglia [1]. Declines in host immunity can reactivation of latent VZV, leading to herpes zoster (HZ), which typically presents as a painful, vesicular dermatomal rash and may be complicated by persistent postherpetic neuralgia (PHN), and severe neurologic or ocular sequelae [2, 3, 4]. Approximately 95% of adults are latently infected with VZV, and about one‐third will develop HZ during their lifetime [5]. With accelerating global population aging, the incidence of HZ continues to rise, resulting in a substantial annual socioeconomic burden [6, 7].

Currently, two vaccines are widely used to prevent HZ: Zostavax (Merck) and Shingrix (GlaxoSmithKline). Zostavax consists of a live attenuated Oka/Merck strain of the VZV, delivered as a single subcutaneous injection. Shingrix is a two‐dose recombinant subunit vaccine based on glycoprotein E (gE) and adjuvanted with AS01B—an adjuvant system composed of QS‐21, monophosphoryl lipid A, and liposomes. However, the real‐world vaccine effectiveness (VE) of Zostavax declines markedly with age and time since vaccination, with VE against HZ falling from approximately 67% in the first year to just 15% after 10 years [8]. Moreover, its live attenuated nature raises safety concerns in immunocompromised individuals [9, 10]. In contrast, two doses of Shingrix confer approximately 74% VE against HZ and 84% against PHN in adults aged ≥ 50 years, with stable efficacy maintained over 4 years [11]. Nevertheless, Shingrix is associated with a higher incidence of local and systemic adverse reactions [12]. Specifically, 17% of Shingrix recipients experience symptoms that hinder normal daily activities (classified as grade 3 adverse reactions), and 1.1% report serious adverse events [12]. Real‐world evidence suggests that tolerability is an important determinant of vaccine uptake. In 2023, only 17% of US adults aged ≥ 50 years completed the full two‐dose series, whereas 4% received only one dose. Among unvaccinated individuals, 46% reported declining vaccination because of concerns about side effects, and among those who received only one dose, 39% cited tolerability concerns as the reason for not completing the series [13]. Moreover, the effects of negative vaccination experiences may extend beyond HZ vaccine uptake itself. In older adults, individuals who received influenza and shingles vaccines on the same day had 0.74 times the odds of receiving an influenza vaccine in a subsequent season, suggesting that adverse vaccination experiences may also influence acceptance of other recommended vaccines [13, 14]. In addition, QS‐21 is limited by supply constraints, safety concerns, and hemolytic activity [15]. Therefore, developing a highly effective HZ vaccine with improved tolerability remains an important public health priority.

Previous studies have shown that cellular immune responses, particularly the VZV‐specific CD4 T‐cell response, plays a critical role in preventing HZ [16]. Accordingly, current HZ vaccine development emphasizes eliciting robust VZV‐specific cellular immunity. In this context, mRNA vaccine technology shows considerable potential. The success of COVID‐19 mRNA vaccines demonstrated that platform's capacity to stimulate potent humoral immune responses while efficiently activating antigen‐specific T‐cell immunity through endogenous antigen presentation [17, 18]. Moderna's VZV mRNA vaccine candidate, mRNA‐1468, induced strong antigen‐specific T‐cell responses and showed favorable tolerability in Phase I/II trials, further supporting mRNA as a promising platform for next‐generation HZ vaccines [19, 20].

Rational antigen selection and design are critical to vaccine performance. Glycoprotein E (gE), the most abundant glycoprotein on the VZV virions surface, mediates viral entry and cell‐to‐cell spread. As a major target of neutralizing antibodies and T‐cell responses, gE has become a key antigen in most HZ vaccine candidates [21]. Notably, in individuals vaccinated with Zostavax, glycoprotein I (gI) also elicits robust CD4 T‐cell responses [22]. The gI is the second most abundant glycoprotein on the VZV virions surface and forms a functional heterodimer with gE. The gE/gI heterodimer contributes to viral dissemination, immune evasion, and Fc receptor‐like activity [23, 24, 25, 26]. Therefore, a dual‐antigen strategy combining gE and gI may further broaden and strengthen vaccine‐induced immunity by increasing T‐cell epitope diversity and better recapitulating native antigen conformation. We have demonstrated that the fusion construct protein gE‐gI, comprising the extracellular domains of gE and gI formulated with the adjuvant XUA09C, induced stronger cellular immune responses than Shingrix in mice, highlighting the superior immunogenicity of the combination of gE and gI [27].

Based on the prior work, this study pursues two primary objectives. First, we systematically evaluated the immunogenicity of mRNAs encoding different combinations and formats of gE and gI, aiming to identify the antigen constructs that induce optimal humoral and cellular immune responses. Second, given that the Toll‐like receptor 9 agonist CpG oligodeoxynucleotides (CpG‐ODNs) can effectively promote Th1 polarization and synergistically enhance humoral and cellular immune responses [28, 29, 30], we further assessed their adjuvant effects with the lead antigen candidate, focusing on their potential to strengthen Th1‐biased cellular immunity. We then evaluated HZ mRNA vaccines based on optimized gE and gI antigen combinations with CpG‐ODN adjuvant for high immunogenicity and safety. Collectively, these efforts inform the rational design of a next‐generation HZ mRNA vaccine by integrating antigen optimization with adjuvant selection.

## 2 Results

### 2.1 Design, Preparation, and Characterization of VZV mRNA Vaccine

We designed a panel of mRNA constructs to compare different antigen presentation formats, including soluble and membrane‐localized gE, soluble and membrane‐localized gEgI fusion proteins, and co‐delivered membrane‐localized gE and gI intended to mimic the native gE/gI heterodimer. Full‐length gE is a 623‐amino acid transmembrane protein containing a signal peptide (SS), a long N‐terminal extracellular domain, a short transmembrane region, and a C‐terminal cytoplasmic region. The extracellular domain mediates binding to insulin‐degrading enzyme (IDE) on the cell surface and represents the main antigenic target for VZV‐specific antibodies and T cells [31], while the C‐terminal domain is critical for subcellular localization [32, 33]. Studies have shown that mRNA vaccines encoding full‐length or truncated gE can elicit both humoral and cellular immunity [34]. In this study, we generated a soluble gE extracellular domain construct (gE_ED_, amino acids 1–537), which is similar to the antigen of Shingrix, and a membrane‐localized gE construct (gE, amino acids 1–573) harboring a Y569A substitution. This substitution alters the trans‐Golgi network (TGN) localization motif from AYRV to AARV, thereby modulating intracellular trafficking and enhancing cell‐surface expression of gE (Figure 1A). Full‐length gI is a 354‐amino acid transmembrane protein. To incorporate gI into tandem antigen designs, we further generated two fusion constructs: gEgI_ED_, a soluble fusion protein formed by linking gE_ED_ to the extracellular domain of gI (gI_ED_, amino acids 21–271) via a (GGGGS)_4_ flexible linker, and gEgI_TM_, a membrane‐localized fusion protein formed by linking gE_ED_ to a membrane‐localized truncated gI construct (gI_TM_, amino acids 21–354) via a (GGGGS)_4_ flexible linker [19, 32, 34] (Figure 1A). To better mimic the native gE/gI heterodimer, we generated a full‐length gI construct (gI, amino acids 1–354) and co‐delivered it with membrane‐localized gE using two strategies, Rmix and Lmix (Figure S1). In the Rmix approach, mRNAs encoding gE and gI were mixed before co‐encapsulation into lipid nanoparticles (LNPs), whereas in the Lmix strategy, each mRNA was encapsulated separately and the resulting LNPs were subsequently combined (Figure S1). These approaches yielded the gE+gI Rmix and gE+gI Lmix formulations, respectively.

**FIGURE 1:**
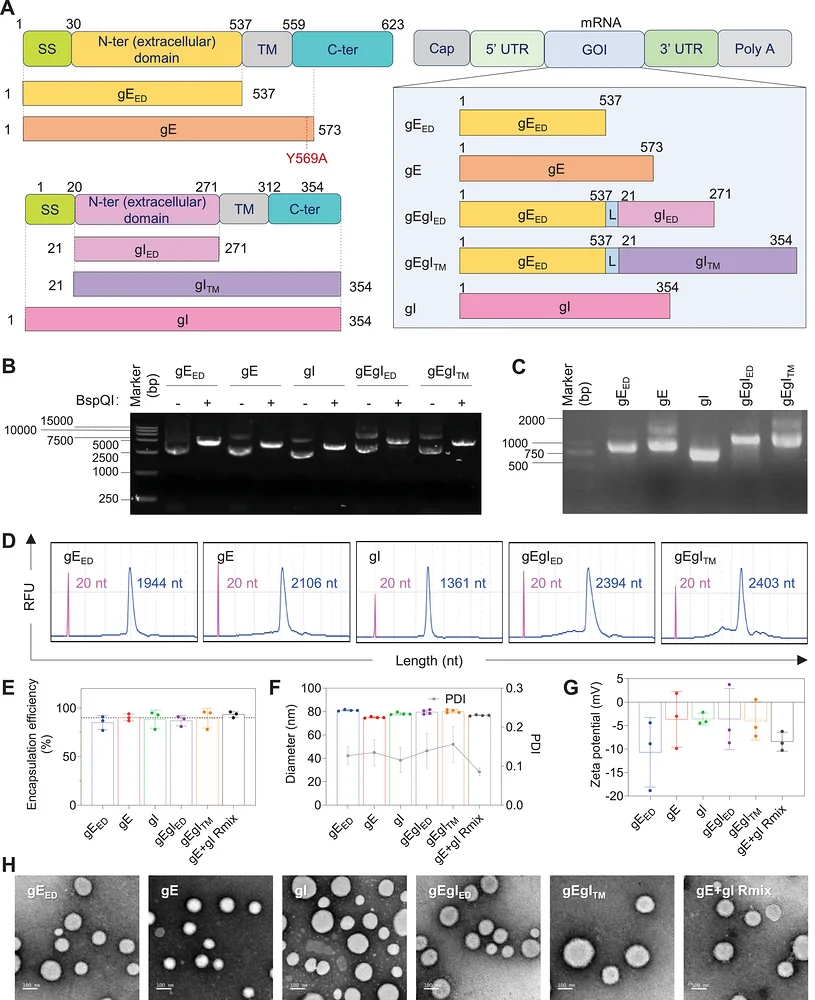
Characterization of VZV mRNA vaccines. (A) Schematic diagram of five antigen designs. SS: signal sequence; L: linker, (GGGGS)_4_; gE_ED_, the extracellular domain of glycoprotein E, amino acids 1–537; gE, a membrane‐localized glycoprotein E harboring a Y569A substitution, amino acids 1–573; gI_ED_, the extracellular domain of glycoprotein I, amino acids 21–271; gI_TM_, a membrane‐localized truncated glycoprotein I, amino acids 21–354; gI, full‐length glycoprotein I, amino acids 1–354; gEgI_ED_: fusion of gE_ED_ and gI_ED_ via a (GGGGS)_4_ linker; gEgI_TM_: fusion of gE_ED_ and gI_TM_ via a (GGGGS)_4_ linker. Each modified mRNA molecule comprises a 5′‐cap, optimized 5′ and 3′ UTRs, a GOI, and a poly A tail. UTR, untranslated regions; GOI, gene of interest. (B) Agarose gel electrophoresis of linearized DNA templates. The DNA size marker is labeled on the left. bp, base pair. (C) Agarose gel electrophoresis of mRNAs synthesized through in vitro transcription. The DNA size marker is labeled on the left. bp, base pair. (D) Capillary electrophoresis of mRNAs. The purple peak represents the RNA Lower Marker, with a size of 20 nt. The blue peak represents individual mRNA, with respective size annotated in blue text. nt, nucleotide. (E) Encapsulation efficiency of mRNA‐LNPs. (F and G) Diameter, polydispersity index (PDI), and zeta potential of mRNA‐LNPs. (H) Representative transmission electron microscopy (TEM) images of mRNA‐LNPs. Scale bar, 100 nm.

Following plasmid construction, highly purified linearized DNA templates were used for in vitro transcription to synthesize corresponding mRNA (Figure 1B). The mRNA purity and integrity were confirmed by agarose gel and capillary electrophoresis (Figure 1C,D). Immunofluorescence in dendritic cells (DC 2.4) cells verified protein expression from each mRNA (Figure S2). The mRNAs were subsequently encapsulated in LNPs to produce vaccines. All mRNA‐LNPs exhibited high encapsulation efficiency, approximately 90% (Figure 1E). Dynamic light scattering (DLS) showed uniform particle size distributions across all mRNA‐LNPs, with mean diameters ranging from 75.0 to 81.1 nm, and polydispersity index (PDI) below 0.3 (Figure 1F). Zeta potentials remained near‐neutral to weakly negative, ranging from −19 to 4 mV (Figure 1G). Transmission electron microscopy (TEM) further confirmed regular spherical morphology of mRNA‐LNPs (Figure 1H). Collectively, we successful produced mRNA vaccines with consistent physicochemical properties, enabling subsequent immunogenicity studies.

### 2.2 Membrane‐Localized gE Construct Glycoprotein E (gE) Induces Robust and Durable Humoral Immunity

To assess the immunogenicity of the VZV mRNA vaccines, mice in seven groups (gE_ED_, gE, gEgI_ED_, gEgI_TM_, gE+gI Rmix, gE+gI Lmix, and PBS control) were immunized intramuscularly (IM) with a 10 µg prime dose, followed by a booster at 3 weeks (Figure 2A). Serum was collected to quantify antigen‐specific IgG antibody titers by enzyme‐linked immunosorbent assay (ELISA). To evaluate the contribution of gI to humoral immune response, we expressed and purified the gE_ED_, gI_ED_, and gEgI_ED_ protein to quantify gE‐, gI‐, or gEgI‐specific binding antibodies induced by vaccines (Figure 2B–D). All vaccines elicited consistent gE‐ and gEgI‐specific binding antibodies levels and trends. The gE, gE+gI Rmix, and gE+gI Lmix vaccines induced the highest gE‐ and gEgI‐specific IgG titers (∼6‐Log), while gEgI_ED_ and gEgI_TM_ vaccines generated comparable IgG titers that were superior to gE_ED_ but lower than the three leading candidates (Figure 2B,D). Only vaccines containing the antigen gI induced gI‐specific IgG antibodies. The gE+gI Rmix elicited the highest gI‐specific IgG titers, followed by gE+gI Lmix, whereas gEgI_ED_ and gEgI_TM_ induced similar but lower gI‐specific titers (Figure 2C). Given the consistent correlation among gE‑ and gEgI‑specific IgG titers, subsequent analyses used gE‑specific IgG titer as the primary humoral readout. Longitudinal monitoring showed that mice immunized with gE, gE+gI Rmix, or gE+gI Lmix vaccines maintained high gE‑specific IgG titers (∼5‐Log) for at least 17 weeks post‑prime, indicating that these mRNA vaccines elicit durable humoral immunity (Figure 2E).

**FIGURE 2:**
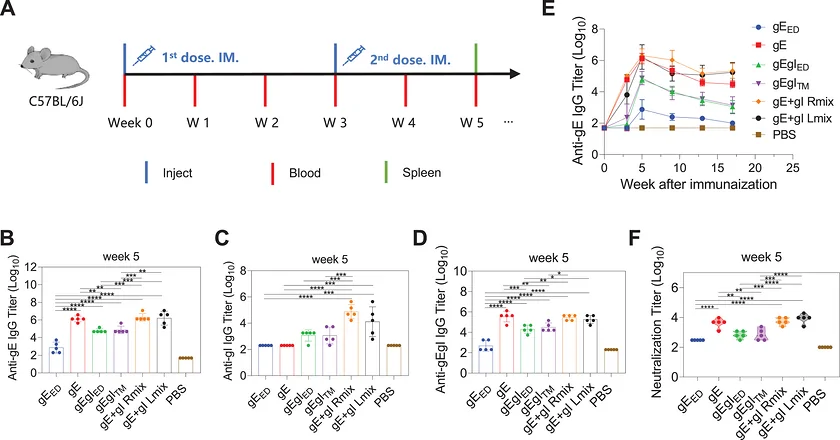
Humoral immune responses induced by VZV mRNA vaccines in mice. (A) Immunization schedule. Female C57BL/6J mice (aged 6–7 weeks, *n* = 5 per group) were immunized intramuscularly with two 10 µg/100 µL doses of the mRNA vaccine. IM, intramuscular. (B–D) The gE‐, gI‐, and gEgI‐specific IgG antibody titers at week 5 measured by endpoint ELISA. (E) The gE‐specific IgG titers were monitored for 17 weeks. (F) Neutralization antibody titers at week 5. Data are presented as means ± SD. Significant differences between groups were analyzed by one‐way ANOVA with Tukey's multiple comparisons test: *p < 0.05; **p < 0.01; ***p < 0.001; ****p < 0.0001.

Neutralizing antibody titers mirrored the gE‐ and gEgI‐specific IgG antibodies trends. The gE, gE+gI Rmix, and gE+gI Lmix induced comparable and highest neutralizing titers (∼4‐Log), outperforming those elicited by gEgI_ED_ and gEgI_TM_, which in turn elicited higher titers than gE_ED_ (Figure 2F).

Taken together, humoral immune analysis showed that membrane‐localized gE, either alone or co‐delivered with gI, elicits stronger and more durable humoral responses than soluble gE‐based constructs. In contrast, the gEgI_ED_ and gEgI_TM_ fusion constructs provide only modest improvement over gE_ED_.

### 2.3 Glycoprotein I (gI) Predominantly Contributes to the Induction of Cellular Immunity

The protective efficacy of HZ vaccines is primarily mediated by T‑cell immunity. CD4^+^ T cells are essential for optimal protection and function synergistically with CD8^+^ T cells to establish a robust immune defense [35]. To evaluate the cellular immune response of vaccines, spleens were harvested from mice 2 weeks after the booster and processed into single‑cell suspensions. Splenocytes were stimulated with peptide pools spanning gE, gI, or gE+gI to assess the contribution of gI to the T‑cell response. Inactivated vOka virus was included as a positive control to mimic whole‐virus antigen stimulation. Antigen‑specific cytokine responses were subsequently quantified.

Enzyme Linked Immunospot (ELISPOT) analysis showed that stimulation with the gE+gI peptide pool elicited the strongest IFN‐γ^+^ and IL‐2^+^ responses (Figure 3A–D, Figure S3A–D). Comparison of the numbers of IFN‐γ^+^ spot‐forming cells (SFC) to individual gE, or gI peptide pools indicated that gI contributed a substantially greater share of the total cytokine response, implying that gI makes a substantial contribution to vaccine‐induced cellular immunity (Figure 3A–C). Moreover, the response trend stimulated with gE+gI peptide pool most closely matched that induced by inactivated vOka virus, suggesting that the combined peptides stimulation better recapitulates virus‑like antigen presentation than the gE or gI peptide pool alone (Figure 3C,D). Among the vaccine groups, gE+gI Rmix and gE+gI Lmix induced the highest numbers of IFN‐γ^+^ and IL‐2^+^ spot‐forming cells (SFCs) following stimulation with either the gE+gI peptide pool or inactivated vOka virus, and these responses were significantly greater than those induced by gE_ED_, gE, gEgI_ED_, and gEgI_TM_ (Figure 3C,D, Figure S3C,D). The gEgI_ED_ and gEgI_TM_ vaccines also produced more IFN‐γ^+^ SFC than gE_ED_ and gE, further supporting a contribution of gI to cellular immunity (Figure 3C,D).

**FIGURE 3:**
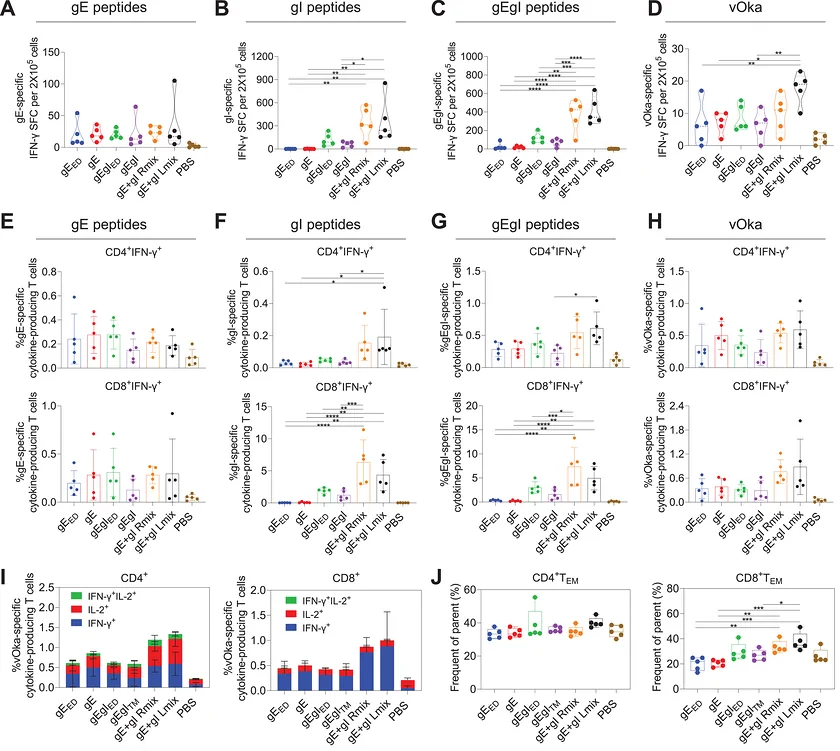
Cellular immune responses induced by VZV mRNA vaccines in mice. (A) The quantification of IFN‐γ^+^ spot‐forming cells (SFC) for splenocytes in mice post‐stimulation with gE, (B) gI, (C) gE+gI peptide pools, (D) or vOka strains by Enzyme Linked Immunospot (ELISPOT) assay. (E) The frequency of cytokine CD4^+^, CD8^+^ IFN‐γ^+^ T cells at week 5 for splenocytes in mice post‐stimulation with gE, (F) gI, (G) gE+gI peptide pools, (H) or vOka strains by flow cytometry. (I) The frequency of CD4^+^, CD8^+^IFN‐γ^+^, or IL‐2^+^, or IFN‐γ^+^IL‐2^+^ T cytokine for splenocytes in mice post‐stimulation with vOka strains by flow cytometry. (J) Long‐living memory T cells by flow cytometry. TEM, effective memory T cell. Data are expressed as means ± SD. Significant differences between groups were analyzed by one‐way ANOVA with Tukey's multiple comparisons test: *p < 0.05; **p < 0.01; ***p < 0.001; ****p < 0.0001.

Consistent with ELISPOT results, flow cytometric showed that cytokine responses to the gE+gI peptide pool mirrored those induced by inactivated vOka virus. Stimulation with either inactivated vOka virus or the gE+gI peptide pool produced the highest frequencies of CD4^+^IFN‐γ^+^ and CD8^+^IFN‐γ^+^ T cells in the gE+gI Rmix and gE+gI Lmix groups (Figure 3E–H, Figure S4A). A similarly elevated IFN‐γ^+^ T‐cell response was observed in these two groups following stimulation with the gI peptide pool alone. In contrast, following stimulation with the gE peptide pool, IFN‐γ^+^ T‐cell frequencies in the gE+gI Rmix and gE+gI Lmix groups were comparable to those in the other vaccine groups, indicating that the enhanced IFN‐γ response is primarily attributable to gI (Figure 3E–G). Moreover, stimulation with inactivated vOka virus or the gE peptide pool yielded the highest frequencies of CD4^+^IL‐2^+^ T cells in the gE+gI Rmix and gE+gI Lmix groups; however, after stimulation with the other peptide pools, all vaccine groups exhibited comparable frequencies of CD4^+^IL‐2^+^ and CD8^+^IL‐2^+^ T cells (Figure S3E–H). Overall, following stimulation with vOka virus, all vaccine groups induced T‐cell responses secreting either IFN‐γ or IL‐2, or both cytokines. Among them, the gE+gI Rmix and gE+gI Lmix groups exhibited the strongest cytokine responses (Figure 3I).

Furthermore, during antigen re‑exposure in the secondary immune response, memory T cells undergo rapid expansion and mount a more effective and faster immune response compared to the primary response [36]. The gE+gI Rmix and gE+gI Lmix also induced the formation of CD8^+^ effective memory T cells (T_EM_) in spleens, contributing to the development of long‐lived memory T cells (Figure 3J, Figure S4B).

In summary, the gE+gI Rmix and gE+gI Lmix groups elicited the most robust T‐cell responses, surpassing those of the gE_ED_ and gE vaccines, indicating that gI enhances vaccine‐induced T‐cell immunity. Furthermore, the co‑delivered antigens induced stronger responses than the fused gEgI_ED_ and gEgI_TM_, possibly because separate antigen expression better preserves native, virus‐like antigen presentation.

### 2.4 CpG‐ODN Adjuvants Effectively Enhance Th1‐Bised Immunity

To further enhance immunogenicity, we formulated the gE+gI Lmix mRNA vaccine with either CpG‐ODN 1018 or CpG‐ODN 2395, and compared resulting formulations with Shingrix. CpG‐ODNs are synthetic immunostimulatory DNA sequences, typically 18–30 base pairs in length. Based on their structural features and immunostimulatory properties, CpG‐ODNs are classified into three types, A, B, and C, which differ in their capacity to stimulate cellular and humoral immune responses. CpG‐ODN 1018 (class B) is the only TLR9 agonist currently approved for use in licensed vaccines, including HEPLISAV‐B and CORBEVAX, whereas CpG‐ODN 2395 (class C) is widely used in vaccine research [37, 38, 39]. CpG‐ODN was added separately to the pre‐formulated gE+gI Lmix mRNA vaccine. Particle size, PDI, and zeta potential measurements showed slight shifts following CpG‐ODN incorporation. However, the changes lacked a consistent directional trend and did not reach statistical significance (Figure S5A–C). Collectively, these results indicate that CpG‐ODN co‐delivery does not substantially alter the key physicochemical parameters of the mRNA‐LNP formulation. Mice were immunized intramuscularly with 10 µg of vaccine co‑administered with 10 µg of CpG‑ODN, following the schedule as described previously (Figure 2A). The 10 µg dose of CpG‐ODN was selected based on the effective CpG‐ODN dosing range established in murine vaccine studies across multiple platforms [40, 41, 42], together with our prior experience demonstrating that this dose effectively potentiates the immunogenicity of the gE‐gI fusion protein combined with the XUA09C adjuvant [27]. As a positive control, a separate group was given 5 µg/50 µL of Shingrix (one‑tenth of the human dose) on the same two‑dose schedule.

Two weeks after the booster, serum was collected to evaluate antibody responses. ELISA results showed that all vaccines produced similar levels of gE‑specific IgG (Figure 4A). However, gE+gI Lmix combined with CpG‑ODN 1018 elicited the highest neutralizing antibody titers, significantly exceeding those of other vaccines, including Shingrix (Figure 4B). All vaccines induced both IgG2c and IgG1 antibodies (Figure 4C). CpG‑ODN 1018 significantly increased the IgG2c/IgG1 ratio, and all CpG‑ODN‑adjuvanted vaccines produced ratios significantly higher than Shingrix (IgG2c/IgG1 > 1), indicating that CpG‑ODN adjuvants promote a Th1‑biased immune response (Figure 4D).

**FIGURE 4:**
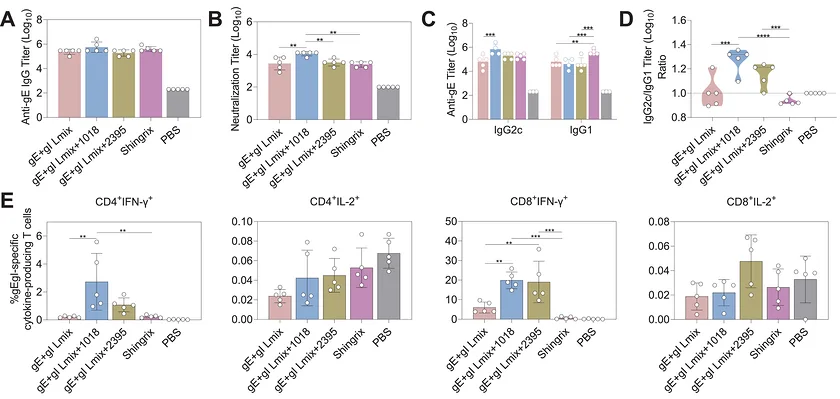
Immune responses of gE and gI dual‐antigen mRNA vaccine adjuvanted with CpG‑ODN. (A) The gE‐specific IgG antibody titers at week 5 by end‐point ELISA. (B) Neutralization antibody titers at week 5. (C) The gE‐specific IgG1 and IgG2c antibody titers at week 5 by end‐point ELISA. (D) The gE‐specific IgG1/IgG2a ratio. (E) The frequency of cytokine CD4^+^, CD8^+^ IFN‐γ^+^, and IL‐2^+^ T cells at week 5 for splenocytes in mice post‐stimulation with gE+gI peptide pools by flow cytometry. Data are expressed as means ± SD. Significant differences between groups were analyzed by one‐way ANOVA with Tukey's multiple comparisons test: *p < 0.05; **p < 0.01; ***p < 0.001; ****p < 0.0001.

To assess antigen‐specific cytokine responses, splenocytes were stimulated with the gE+gI peptide pool, based on our previous results (Figure 3G,H). Compared with Shingrix, the unadjuvanted gE+gI Lmix vaccine elicited a comparable frequency of CD4^+^IFN‑γ^+^ T cells and a higher frequency of CD8^+^IFN‑γ^+^ T cells (Figure 4E). Co‑administration with any CpG‑ODN substantially increased both CD4^+^IFN‑γ^+^ and CD8^+^IFN‑γ^+^ frequencies. The CD8^+^IFN‑γ^+^ levels induced by adjuvanted vaccines significantly exceeded those elicited by either the unadjuvanted gE+gI Lmix vaccine or Shingrix. Notably, the combination with CpG‑ODN 1018 induced the highest CD4^+^IFN‑γ^+^ response (Figure 4E). In contrast to this broad enhancement of IFN‑γ production, IL‑2 responses were more restricted. Only gE+gI Lmix + CpG‐ODN 1018 significantly elevated CD4^+^IL‐2^+^ T cell frequencies above the unadjuvanted vaccine level, whereas CD8^+^IL 2^+^ responses remained comparable across all groups (Figure 4E).

Collectively, CpG‐ODN adjuvants enhanced binding and neutralization antibody titers to varying degrees. In particular, CpG‐ODN 1018 strengthened the Th1‑biased immunogenicity of the gE+gI Lmix mRNA vaccine in mice, surpassing the Shingrix vaccine in key humoral and cellular immune parameters.

### 2.5 gE and gI Dual‐Antigen mRNA Vaccine Adjuvanted With CpG‐ODN Exhibits an Acceptable Preclinical Safety Profile

To evaluate toxicology profiles of mRNA vaccine with and without CpG adjuvant, we monitored body weight for 7 days after immunization and evaluated serum biochemistry and tissue histopathology. Mice immunized with the unadjuvanted VZV mRNA vaccine showed no significant change in body weight compared to PBS controls (Figure S6A,B). Serum levels of alanine aminotransferase (ALT), aspartate aminotransferase (AST), and creatinine (CREA) were comparable across all vaccine groups, indicating a favorable initial safety profile for the mRNA vaccine platform (Figure S6C). We next assessed the safety of CpG‑adjuvanted formulations. Body weight decreased transiently 1–2 days after immunization but recovered to baseline within 7 days (Figure 5A). Area‑under‑the‑curve (AUC) analysis revealed that mice immunized with gE+gI Lmix combined CpG‑ODN 1018 exhibited significantly lower cumulative weight gain than PBS controls and the unadjuvanted gE+gI Lmix vaccine, indicating transient weight loss associated with this combination (Figure 5B). However, serum ALT, AST, and CREA levels remained within normal ranges and showed no significant differences from PBS controls (Figure 5C). Furthermore, histopathology H&E analysis of heart, liver, spleen, lung, and kidney confirmed no notable tissue damage or morphological changes in all vaccinated mice (Figure 5D).

**FIGURE 5:**
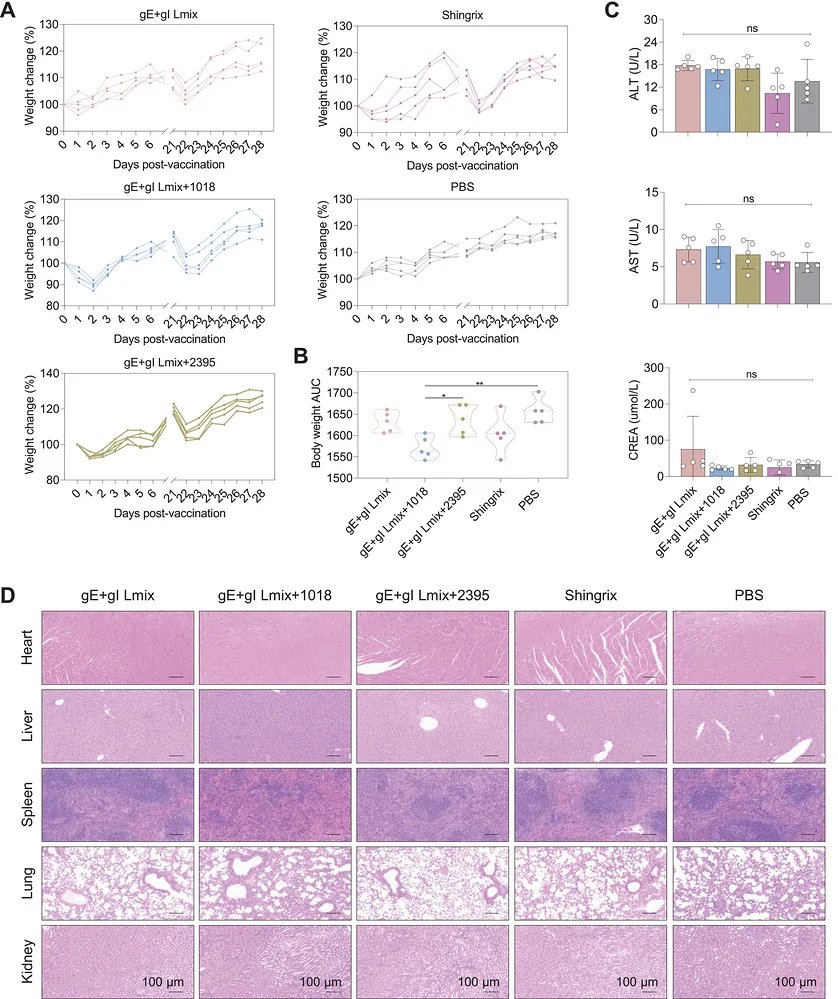
Safety profile of gE/gI dual‐antigen mRNA vaccine adjuvanted with CpG‑ODN in mice. (A) Body weight changes following immunization. (B) Area‑under‑the‑curve (AUC) of body weight changes. (C) Serum biochemical analysis of liver and kidney function at week 5. ALT, alanine aminotransferase, AST, aspartate aminotransferase, indicate liver function; CREA, creatinine, indicates kidney function. (D) Representative histopathology H&E images of heart, liver, spleen, lung, and kidney at week 5. Scale bar  =  100 µm, 10×. Data are expressed as means ± SD. Significant differences between groups were analyzed by one‐way ANOVA with Tukey's multiple comparisons test: *p < 0.05; **p < 0.01.

In summary, while the gE+gI Lmix combined CpG‑ODN 1018 was associated with a transient and quantifiable weight loss, all vaccine candidates demonstrated an acceptable preclinical safety profile with no significant systemic toxicity or histopathology.

### 2.6 gE/gI Dual‐Antigen mRNA Vaccine Adjuvanted With CpG‐ODN Induces Strong Immune Responses of in VZV‐Primed Mouse Model

To model pre‐existing immunity following VZV exposure, mice were primed subcutaneously with the live‐attenuated vOka strain 4 weeks before vaccination. Based on its favorable Th1‐promoting activity in earlier experiments, CpG‐ODN 1018 was selected for further evaluation. Primed mice then received intramuscular immunization with the mRNA vaccine at weeks 0 and 3, while control mice were given vOka priming followed by PBS (Figure 6A).

**FIGURE 6:**
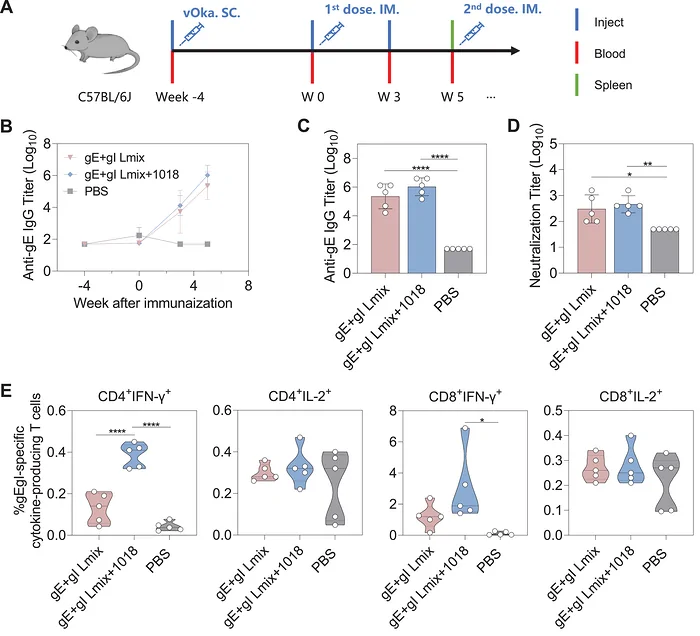
Immunogenicity of VZV mRNA vaccine in VZV‐primed mouse models. (A) Immunization schedule. Female C57BL/6J mice (aged 6–7 weeks, *n* = 5 per group) were primed subcutaneously with 2000 PFU (plaque‐formation units) of the vOka strain in 100 µL, administered 4 weeks before vaccination to establish a VZV‐primed mouse model. Mice were subsequently immunized intramuscularly with two 10 µg/100 µL doses of the mRNA vaccine. IM, intramuscular; SC, subcutaneous. (B) Longitudinal monitoring of gE‐specific IgG titers. (C) The gE‐specific IgG antibody titers at week 5 by end‐point ELISA. (D) Neutralization antibody titers at week 5. (E) The frequency of cytokine CD4^+^, CD8^+^ IFN‐γ^+^, and IL‐2^+^ T cells at week 5 for splenocytes in mice post‐stimulation with gE+gI peptide pools by flow cytometry. Data are expressed as means ± SD. Significant differences between groups were analyzed by one‐way ANOVA with Tukey's multiple comparisons test: *p < 0.05; **p < 0.01; ***p < 0.001; ****p < 0.0001.

Serum analysis showed that a single vaccine dose elicited detectable gE‑specific IgG antibodies, with titers increasing significantly after a booster dose (Figure 6B). At week 5, gE‑specific binding titers and neutralizing antibody levels induced by gE+gI Lmix combined with CpG‑ODN 1018 were slightly higher than those induced by the unadjuvanted gE+gI Lmix vaccine (Figure 6C,D). To assess vaccine‐induced cellular immunity, splenocytes were collected at week 5 for cytokine analysis. Notably, inclusion of CpG‐ODN 1018 significantly enhanced the frequency of CD4^+^IFN‐γ^+^ T cells relative to the unadjuvanted formulation, accompanied by modest increases in CD8^+^IFN‐γ^+^, CD4^+^IL‐2^+^, and CD8^+^IL‐2^+^ T cells (Figure 6E).

Collectively, these results demonstrate that CpG‑ODN 1018 effectively enhance both humoral and cellular immunity in a VZV‐primed mouse models, further supporting their use as potent Th1‐bised adjuvants for HZ mRNA vaccines.

## 3 Discussion

This study systematically develops and evaluates a novel HZ mRNA‐LNP vaccine by combining rational antigen design with adjuvant optimization. We demonstrate that a dual‐antigen mRNA vaccine co‑delivering membrane‐localized VZV gE and gI (gE+gI Lmix), when adjuvanted with CpG‑ODN 1018, elicits a robust, Th1‑biased immune response in mice. Notably, the key preclinical cellular immune metrics, including antigen‐specific CD4^+^IFN‐γ^+^ T‐cell responses, were comparable to or exceeded those induced by the current standard vaccine, Shingrix, in the mouse model, while maintaining an acceptable preclinical safety profile.

Our systematic antigen optimization revealed a clear immunogenicity hierarchy. Membrane‐localized gE elicited stronger and more durable antibodies responses than its soluble extracellular domain gE_ED_, which aligns with previous studies [34]. This advantage is likely attributable to several features of the full‐length membrane‐anchored format. First, the Y569A substitution redirects gE from the TGN to the cell surface, thereby maximizing antigen density on the plasma membrane for immune recognition [32, 33]. Second, membrane anchoring preserves the native conformational context of gE, including transmembrane‐proximal epitopes and glycosylation patterns that are lost in the truncated soluble gE_ED_. These structural features may be critical for generating conformation‐dependent neutralizing antibodies. In contrast, gI contributes more prominently to cellular than to humoral immunity, consistent with reports that gI elicits broad CD4^+^ T‐cell responses in Zostavax‐vaccinated individuals [22]. Strikingly, the co‐delivery of separate gE and gI mRNAs (Rmix/Lmix) consistently elicited stronger T‑cell responses than genetically fused constructs gEgI_ED_ and gEgI_TM_. Because gE and gI naturally form a non‐covalent heterodimer [24, 25], separate expression may allow each protein to fold and undergo post‐translational modification independently, while retaining the potential to associate through its native interaction interfaces and broadening immune recognition. The gE is a major target of antibody responses, whereas gI may broaden T‐cell recognition. Co‐delivery could therefore expand the range of antigens available to the immune system and support neutralizing antibodies production and cellular responses. Additionally, the mRNA platform may further contribute by endogenous antigen expression and presentation through MHC class I pathways, while antigen uptake and processing by antigen‐presenting cells can support MHC class II presentation. Together, these findings may help explain the strong immunogenicity of the gE+gI Lmix formulation. Further studies are needed to establish the extent of gE/gI heterodimer formation and to define the antigen‐presentation mechanisms underlying these responses.

To further enhance vaccine immunogenicity, we incorporated CpG‐ODN adjuvants into the mRNA vaccine formulation. Among the candidates tested, CpG‑ODN 1018 most clearly promoted a Th1‐biased response, as evidenced by an increased IgG2c/IgG1 ratio and enhanced IFN‑γ production by both CD4^+^ and CD8^+^ T cells. The synergy may reflect complementary innate immune stimulation by the mRNA vaccine and TLR9 agonist CpG‐ODN, potentially promoting dendritic cell maturation and Th1 differentiation [43]. In mice, the gE+gI Lmix combined CpG‐ODN 1018 amplified antibody and IFN‐γ^+^ T cell levels that significantly exceeded those induced by Shingrix, underscoring the potential of this combinatorial approach. It should be noted that the CpG‐ODN was administered as a simple admixture with mRNA‐LNP rather than co‐encapsulated within the LNP. Co‐encapsulation of CpG‐ODN with the mRNA payload may offer advantages in terms of co‐delivery to the same antigen‐presenting cells and enhanced TLR9‐mediated innate immune activation, although it introduces additional formulation complexity. The admixture approach used here provides a practical and flexible strategy for dose optimization. Future studies should directly compare admixture and co‐encapsulation strategies to determine the optimal CpG‐ODN delivery method for mRNA‐based HZ vaccines.

An acceptable preclinical safety profile is essential for vaccine development. The candidate formulations showed no evidence of marked systemic toxicity or histopathological damage in the organs examined. The main observed effect was a transient reduction in body weight in mice receiving gE+gI Lmix with CpG‑ODN 1018. Body weight returned to baseline within 7 days, and no other signs of toxicity were detected [44]. These findings define a manageable reactogenicity window and provide an important basis for future dose‐optimization studies aimed at balancing immunogenicity and tolerability.

Protection against HZ involves a complex immune correlate, necessitating both VZV‐specific antibodies to limit neuronal reinfection and robust polyfunctional T‐cell responses to suppress viral reactivation. Our candidate can elicit neutralizing antibody titers that surpass those induced by Shingrix in mice, alongside robust T‐cell response that amplified by CpG‐ODN 1018. The induction of effector memory T cells (T_EM_) further implies the potential for durable protection. Notably, the vaccine retained high immunogenicity in a VZV‑primed mouse model, supporting its further evaluation as a potential booster for individuals with pre‑existing VZV immunity, which are the primary target population for HZ vaccination.

Several limitations of the current study should be acknowledged. First, due to the strict species specificity of VZV, which infects only humans and a limited range of non‐human primates, there is no readily available small‐animal challenge model to directly assess vaccine‐mediated protective efficacy against viral infection or reactivation. While the mouse model is widely used for preliminary immunogenicity screening, it does not fully recapitulate the natural history of VZV latency or the immunological milieu of human HZ [45]. Consequently, the immune correlates measured in this study represent surrogate endpoints, and their relationship to clinical protection remains to be established in human studies. Second, all experiments were conducted in young adult mice, which do not represent the older adults (≥ 50 years) who are the primary target population for HZ vaccination. Because immunosenescence can affect vaccine responses, studies in aged animal models or non‐human primates would provide more clinically relevant insights. Third, although a head‑to‑head comparison with Shingrix was performed in naïve mice, a parallel Shingrix positive control was not included in the VZV‑primed mouse model due to the limited availability of this commercial vaccine, which prevented a direct comparison under conditions of pre‑existing VZV immunity. Fourth, antibody responses were monitored for up to 17 weeks after priming, whereas cellular immune responses were assessed over a shorter period. Longer term studies are needed to characterize the durability of T‐cell memory. Finally, the safety assessments were limited to short‐term preclinical observations, and further studies should assess local reactogenicity and long‐term safety.

Despite these limitations, our findings provide a strong foundation for further optimization and translational development of this promising HZ vaccine candidate. Overall, we have developed a novel HZ vaccine candidate that merges three advanced vaccinology principles: (1) an mRNA platform for potent endogenous antigen presentation, (2) a rationally designed dual‐antigen strategy to recapitulate native viral immunogenicity, and (3) a synergistic Th1 adjuvant to amplify and polarize the response. The resulting gE+gI Lmix combined CpG‐ODN 1018 vaccine induces a comprehensive immune profile matches or exceeds the licensed vaccine Shingrix in mice and exhibits an acceptable preclinical safety profile. This study provides a promising strategy for developing a next‐generation HZ vaccine with the potential for higher efficacy.

## 4 Materials and Methods

### 4.1 Cells, Virus, Vaccines, and Antibodies

Human acute retinal epithelial cells, ARPE‐19 (ATCC), were cultured in DMEM/F12 (1:1) medium (Thermo Fisher Scientific) supplemented with 10% fetal bovine serum (FBS) (PANBiotech) to measure neutralization titers. Dendritic cells, DC 2.4 cells (ATCC), were cultured in RPMI 1640 medium (BasalMedia) with 2% FBS for transient transfection to detect mRNA expression through immunofluorescence. Human embryonic kidney cells, HEK293F, were maintained in OPM‐293 CD05 Medium (Shnghai OPM Biosciences) for protein expression. Regular mycoplasma testing was conducted on all cell lines, confirming their mycoplasma‐free status. Immunofluorescence analysis was conducted using anti‐gE (4A2) and anti‐gI (9D11) monoclonal antibodies produced in our laboratory. The vOka strain (Oka/Merck strain) of VZV was provided by Beijing Wantai Biological. The Shingrix vaccine (GlaxoSmithKline) served as a control. Vaccine adjuvants, CpG‐ODN 1018 (5′‐TGACTGTGAACGTTCGAGATGA‐3′) and CpG‐ODN 2395 (5′‐TCGTCGTTTCGGCGCGCGCCG‐3′), were purchased from InvivoGen. Both CpG‐ODNs were of VacciGrade quality.

### 4.2 Production and Purification of mRNA

DNA templates for in vitro transcription were prepared by linearizing plasmids with the restriction enzyme BSPQI (New England Biolabs). Modified mRNA was then synthesized using the T7 High Yield RNA Transcription Kit (Vazyme) and linear DNA templates. The transcription substituted UTP with N1‐Me‐Pseudo‐UTP (CANGZHOU VACCINES) and included cap analog 7MeG(3′Ome)‐5′‐ppp‐5′‐A(OMe)G (CANGZHOU VACCINES) for co‐transcriptional capping. The mRNA was purified via lithium chloride precipitation and subsequently resuspended in RNase‐free water, and quantified using Quant‐iT RiboGreen RNA Kit (Thermo Fisher Scientific).

### 4.3 LNP Encapsulation of mRNA

The mRNA was encapsulated in LNP using the same lipid composition as the BNT162b2 vaccine [46]. Briefly, lipids (ALC‐0315, ALC‐0159, DSPC, and cholesterol) were dissolved in ethanol at a molar ratio of 46.3:1.6:9.4:42.7. The lipid mixture was combined with mRNA in 50 mM citrate buffer (pH 3.0) (Macklin) at a 1:3 volume ratio using a T‐mixer. Then, the formulation was immediately diluted 10‐fold in phosphate buffered saline (PBS) (pH 7.4) and processed by a 10 kDa tangential flow filtration membrane for buffer exchange and concentration. The particle size and zeta potential of mRNA‐LNP was measured by dynamic light scattering (DLS). The morphology was analyzed by transmission electron microscopy (TEM) using the negative staining technique. The mRNA encapsulation efficiency was detected using Quant‐iT RiboGreen RNA Kit (Thermo Fisher Scientific) by calculating the ratio of encapsulated mRNA (total minus free) to total mRNA.

### 4.4 Immunofluorescence

DC 2.4 cells were seeded into six‐well plates at a density of 1 × 10^6^ cells per well and transfected with 10 µg of mRNA, specifically 10 µg gE_ED_, gE, gI, gEgI_ED_, gEgI_TM_, using Lipofectamine 3000 Transfection Reagent (Thermo Fisher Scientific). Twenty‐four hours post‐transfection, the cells were fixed in a 4% paraformaldehyde solution (BEYOTIME) at room temperature for 20 min and permeabilized with 2% Triton X‐100 in PBS. The cells were then blocked with PBS containing 2% FBS and incubated with either the anti‐gE antibody (4A2) or the anti‐gI antibody (9D11) at room temperature for 30 min. The cells were then washed with PBS and incubated with an Alexa Fluor 647 Donkey Anti‐Mouse IgG (H+L) secondary antibody (Thermo Fisher Scientific) at room temperature for 30 min. After washing, a cell membrane dye, Dio (BEYOTIME), was applied and incubated at room temperature for 20 min, followed by five additional washes with PBS. Finally, the cells were mounted on slides using DAPI mounting medium (BEYOTIME).

### 4.5 Expression and Purification of Proteins

We express and purificate the soluble ectodomains of gE_ED_ (a soluble form comprising the extracellular domain of full‐length gE, amino acids 1–537), gI_ED_ (a soluble form comprising the extracellular domain of full‐length gI, amino acids 21–271), and gEgI_ED_ (a construct by linking gE_ED_ to gI_ED_ via a (GGGGS)_4_ flexible linker) to quantify gE‐, gI‐, and gEgI‐specific binding antibodies, respectively. The ectodomain proteins were selected because they represent the conformationally relevant extracellular regions of the gE, gI, and gE/gI complex, which are the primary targets of neutralizing antibodies, while avoiding the technical challenges of full‐length transmembrane protein purification. The genes encoding gE_ED_, gI_ED_, and gEgI_ED_ with a C‑terminal 6×His tag were synthesized and cloned into the pcDNA3.4 vector. Each plasmid was transiently transfected into HEK293F cells. After 6 days, the secreted proteins were purified from the culture supernatant using Ni‑Sepharose Fast Flow 6 resin (Cytiva) and eluted with 250 mM imidazole.

### 4.6 Immunization in Mice

Female C57BL/6J mice (aged 6–7 weeks, *n* = 5 per group) were housed under specific pathogen‐free (SPF) conditions. Mice were immunized intramuscularly (IM) with mRNA‐LNP vaccine candidates (10 µg/100 µL per dose) according to a prime‐boost regimen (two doses administered 3 weeks apart). A positive control group was administered 5 µg/50 µL of the adjuvanted recombinant herpes zoster vaccine (Shingrix, GSK) intramuscularly according to the manufacturer's instructions (Shingrix, INN‐Herpes zoster vaccine (recombinant, adjuvanted)). To establish a VZV‑primed mouse model, female C57BL/6J mice (aged 6–7 weeks) were injected subcutaneously with 2000 plaque‐forming units (PFU) of the vOka strain (Oka/Merck strain, Beijing Wantai Biological) in 100 µL PBS, 4 weeks prior to the first immunization. All animal procedures were approved by the Laboratory Animal Management Ethics Committee of Xiamen University (Approval No. XMULAC20240380) and conducted in accordance with the relevant ethical guidelines.

### 4.7 ELISA

A 96‐well microplate was coated with protein (100 ng/well) and incubated at 37°C for 2 h. Wells were blocked with 1×enzyme dilution buffer (PBS containing 0.25% casein, 1% gelatin, 0.05% proclin‐300) (Merck Sigma‐Aldrich) at 37°C for 2 h. Serial dilutions of serum samples (100 µL per well) were added and incubated at 37°C for 30 min. Then, the plates were washed and treated with secondary antibody (HRP‐conjugated reagent) at 37°C for 30 min. Colorimetric detection was performed with o‐phenylenediamine (OPD) substrate (WANTAI BioPharm, Beijing) at 37°C for 10 min, followed by the addition of stop solution (WANTAI BioPharm, Beijing). Absorbance (450 nm, with a reference wavelength of 620 nm) was measured on a microplate reader (TECAN, Männedorf, Switzerland). The endpoint titers were defined as the highest reciprocal serum dilution yielding an absorbance exceeding the mean background values plus three standard deviations.

### 4.8 Neutralization Assay

The neutralization assay was performed with minor modifications as previously described [27, 47]. Serum samples were heat‐inactivated at 56°C for 30 min and then serially diluted in protection buffer (25 mM histidine, 150 mM NaCl, 9% sucrose, pH 7.4). Diluted sera were incubated with approximately 100 PFU of the vOka strain (WANTAI BioPharm) in protection buffer supplemented with complement (1:10, v/v) at 37°C for 1 h. The mixture was then transferred onto pre‐seeded ARPE‐19 cells in 48‐well plates (NUNC, Roskilde, Denmark) with 70% confluence and incubated for additional 1 h at 37°C. Cells were subsequently maintained in fresh DMEM/F12 medium at 37°C, with mock‐infected ARPE‐19 cells included as a control. After 48–72 h, infection was assessed by ELISPOT assay as previously described [48]. Neutralization titers were defined as the highest serum dilutions that neutralized virus.

### 4.9 ELISPOT Assay

Mice were euthanized humanely and spleens aseptically harvested. Spleens were mechanically dissociated in cold RPMI‑1640 medium (BasalMedia) supplemented with 2% FBS. The resulting cell suspension was centrifuged at 2000 rpm for 5 min, and the supernatant was discarded. The cell pellet was then resuspended in 10 mL of cold red blood cell lysis buffer (Solarbio) and incubated at room temperature for 10 min. The lysis reaction was stopped by adding 20 mL of cold 1640 medium with 2% FBS, followed by centrifugation at 2000 rpm for 5 min. The supernatant was removed, and the purified splenocytes were resuspended in 5 mL of RPMI‑1640 medium with 2% FBS for subsequent analysis.

Splenocytes were seeded 2 × 10^5^ cells per well into pre‑coated anti‐mouse IFN‐γ plates (Dakewe Biotech). Cells were incubated with overlapping antigen peptide pools derived from gE or/and gI for 20 h at 37°C in 5% CO_2_. Spots were enumerated and analyzed using a CTL‐ImmunoSpot S5 analyzer (Cellular Technology Limited) according to the manufacturer's instructions.

### 4.10 T‐Cell Flow Cytometry Analysis

Surface staining for effective memory T cells (T_EM_). Splenocytes were marked with PerCP/Cyanine5.5 anti‐mouse CD8α (clone 53–6.7, Biolegend), FITC anti‐mouse CD4 (clone GK1.5, Biolegend), PE/Cyanine7 anti‐mouse/human CD44 (clone IM7, Biolegend), APC/Cyanine7 anti‐mouse CD62L (clone MEL‐14, Biolegend), and LIVE/DEAD Fixable Aqua Dead Cell Stain Kit (Thermo Fisher Scientific & Invitrogen) for 30 min at 4°C in the dark. Subsequently, cells were washed and resuspended with FACS buffer (PBS containing 2% FBS), and detected on BD LSRFortessa X‐20 Flow Cytometer.

Intracellular cytokine staining (ICS): Splenocytes were incubated with overlapping antigen peptide pools derived from gE or/and gI for 18 h at 37°C in 5% CO_2_. Then, protein transport inhibitor (BD GolgiPlug, BD Biosciences) was added to splenocytes and incubated for 6 h. Splenocytes were washed with FACS buffer and stained using PE/Cyanine7 anti‐mouse CD8α (clone 53–6.7, Biolegend), FITC anti‐mouse CD4 (clone GK1.5, Biolegend), and LIVE/DEAD Fixable Aqua Dead Cell Stain Kit (Thermo Fisher Scientific & Invitrogen) for 30 min at 4°C in the dark. Subsequently, splenocytes were fixed and permeabilized using the Fixation/Permeabilization Solution Kit (BD Biosciences), and then stained with APC anti‐mouse IFN‐γ (clone XMG1.2, BD Biosciences) and PE anti‐mouse IL‐2 (clone JES6‐5H4, BD Biosciences) for 30 min at 4°C in the dark. Finally, splenocytes were washed and resuspended with 1×Perm Wash solution (BD Biosciences), and detected on BD LSRFortessa X‐20 Flow Cytometer.

### 4.11 In Vivo Toxicity

To evaluate the in vivo toxicity of the vaccine, body weights of mice were monitored after each immunization. Serum was analyzed for biochemical markers using assay kits, including alanine aminotransferase (ALT), aspartate aminotransferase (AST), and creatinine (CREA) using Assay Kits (Nanjing Jiancheng Bioengineering Institute). For histopathological analysis, organ tissues, including heart, liver, spleen, lung, and kidney, were harvested and fixed in 4% neutral‐buffered formaldehyde, embedding in paraffin, and sectioned. Sections were stained with hematoxylin and eosin (H&E) and examined under a light microscope (NIKON Eclipse CI).

### 4.12 Statistical Analysis

GraphPad Prism (9.5.1) was utilized for all statistical analyses. Data were presented as means ± standard deviations (SD). Significant differences between groups were analyzed by one‐way ANOVA or two‐way ANOVA with Tukey's multiple comparisons test. Significance levels are denoted as follows: *p < 0.05, **p < 0.01, ***p < 0.001, ****p < 0.0001; “ns” indicates no significant difference. Microsoft Office (2024) was additionally used to assist in the preparation of selected figures, charts, and graphical elements.

## Author Contributions

G.Y., S.L., and T.L. designed the study. L. Cui, S. Z., H. Liu, W. X., H. W., H. Li, Y.Z., H.Z., Y.L., M.H., and L. Chen performed experiments. L. Cui, S.Z., H. Liu, Y.G., S.L. T.L., and N.X. analyzed data. L. Cui, T.L., and S.L. wrote the manuscript. L. Cui, S.Z., H. Liu, J.Z., Y.G., S.L. T.L., and N.X. participated in discussion and interpretation of the results. All authors contributed to experimental design. All authors have read and approved the final manuscript.

## Funding

This work was supported by grants from the National Key Research and Development Program of China (grant no. 2023YFC2307602‐,2024YFC2310400), the National Natural Science Foundation (grant no. 82471861), and the Fundamental Research Funds for the Central Universities (grant no. 20720250004).

## Ethics Statement

All animal procedures were approved by the Laboratory Animal Management Ethics Committee of Xiamen University (Approval No. XMULAC20240380) and conducted in accordance with the relevant ethical guidelines.

## Conflicts of Interest

T.L., S.L., S.Z., Y.Z., L. Cui, Y.G., T.C., and N.X. are inventors on a patent application (PCT/CN2024/073289) filed by the Xiamen University that covers gEgI_ED_ fusion protein design described in this work. The remaining authors declare no conflicts of interest.

## Supporting information




**Supporting File**: mco271117‐sup‐0001‐SuppMat.docx

## Data Availability

All data are available in the main text or the Supplementary Information.
