# Supplementary material for: Co‐Delivery of Varicella Zoster Virus gE and gI Dual‐Antigen mRNA Vaccine With CpG‐ODN Elicits Robust Immunity
Source: MedComm (2020). 2026 Nov 1;7(11):e71117. doi: 10.1002/mco2.71117 (PMC13725205; doi:10.1002/mco2.71117)
Supplement: Supplementary file 1 [file MCO2-7-e71117-s001.docx]

**Co-delivery of** **Varicella Zoster Virus** **gE and gI Dual-antigen mRNA Vaccine with CpG-ODN Elicits Robust Immunity**

Lingyan Cui^1,2#^, Sibo Zhang^1,2#^, Wenhui Xue^1,2#^, Hongjing Liu^1,2#^, Hong Wang^1,2^, Hai Li^1,2^, Yarong Zeng^1,2^, Huizheng Zhang^1,2^, Yaohan Li^1,2^, Minqing Hong^1,2^, Lin Chen^1,2^, Jun Zhang^1,2^, Ying Gu^1,2*^, Shaowei Li^1,2*^，Tingting Li^1,2*^, Ningshao Xia^1,2^

^1^ State Key Laboratory of Vaccines for Infectious Diseases, Xiang An Biomedicine Laboratory, School of Public Health, School of Life Sciences, Xiamen University, Xiamen 361102, China

^2^ National Institute of Diagnostics and Vaccine Development in Infectious Diseases, Collaborative Innovation Center of Biologic Products, National Innovation Platform for Industry-Education Integration in Vaccine Research, the Research Unit of Frontier Technology of Structural Vaccinology of Chinese Academy of Medical Sciences, Xiamen University, Xiamen 361102, China

^#^ These authors contributed equally to this work.

* Correspondence: [guying@xmu.edu.cn](mailto:guying@xmu.edu.cn) (Y. G.); [shaowei@xmu.edu.cn](mailto:shaowei@xmu.edu.cn) (S.L.) and [litingting@xmu.edu.cn](mailto:litingting@xmu.edu.cn) (T.L.)

**Fig S1. Co‑delivery strategies of mRNAs.** For the Rmix approach, different mRNAs are mixed before co‑encapsulation in lipid nanoparticle (LNP). For the Lmix approach, different mRNAs are individually encapsulated, and the respective LNPs were mixed.

**Fig S2. Representative immunofluorescence analysis demonstrating expression of mRNA encoding antigens by DC 2.4 cells *in vitro*.** Blue staining indicates DAPI-stained nuclei, and green staining indicates DIO-stained cell membranes. DyLight 650-tagged monoclonal antibodies, anti-gE (4A2), anti-gI (9D11) indicate gE- or gI-positive cells. Merged images reveal the co-localization of antigens with DC 2.4 cells. Scale bars coloured in white, 10 μm.

**Fig S3.** **The VZV mRNA vaccines elicit robust cellular immune response in mice.** (**A**) The quantification of IL-2^+^ spot-forming cells (SFC) for splenocytes in mice post-stimulation with gE, (**B**) gI, (**C**) gE+gI peptide pools, (**D**) or vOka strains by ELISPOT. (**E**) The frequency of cytokine CD4^+^, CD8^+^ IL-2^+^ T cells at week 5 for splenocytes in mice post-stimulation with gE, (**F**) gI, (**G**) gE+gI peptide pools, (**H**) or vOka strains by flow cytometry. Data are expressed as means ± SD. Significant differences between groups were analyzed by one-way ANOVA with Tukey’s multiple comparisons test *: p < 0.05; **: p < 0.01; ***: p < 0.001; ****: p < 0.0001.

**Fig S4. Gating strategy for flow cytometry analysis.** (**A**) Gating strategies for anrigen-specific cytokine-secreting CD4+/CD8+ IFN-γ+/IL-2+ T cells. (**B**) Gating strategies for CD4+/CD8+ T_EM_ (CD44+CD62L^-^) cells.

**Fig S5. Characterization of mRNA-LNPs combined with CpG‑ODN.** (**A**) Diameter, (**B**) polydispersity index (PDI), (**C**) and zeta potential of mRNA-LNPs. Data are expressed as means ± SD. Significant differences between groups were analyzed by two-way ANOVA with Tukey’s multiple comparisons test. “ns” indicates no significant difference.

**Fig S6. Safety evaluation of VZV mRNA vaccine in mice.** (**A**) Body weight changes after administrations of vaccines within 7 days. (**B**) (**C**) Levels of plasma pro-inflammatory cytokines are measured 48 h after initial administration of vaccines. (**D**) Liver and kidney function are determined at week 5 (2 weeks after the 2nd boost) by blood biochemical parameters. ALT, alanine aminotransferase, AST, aspartate aminotransferase, represent liver function, and CREA, creatinine, represents kidney function. Data are expressed as means ± SD. Significant differences between groups were analyzed by one-way ANOVA with Tukey’s multiple comparisons test.
